# Awareness of HPV Testing and Acceptability of Self-sampling for Cervical Cancer Screening Among Women in Minnesota

**DOI:** 10.1007/s11606-021-06854-x

**Published:** 2021-05-13

**Authors:** Xuan Zhu, Kathy L. MacLaughlin, Chun Fan, Debra J. Jacobson, Gregory D. Jenkins, Robert M. Jacobson, Lila J. Finney Rutten

**Affiliations:** 1 Robert D. and Patricia E. Kern Center for the Science of Health Care Delivery, Mayo Clinic, Rochester, MN USA; 2 Department of Family Medicine, Mayo Clinic, Rochester, MN USA; 3 Department of Quantitative Health Sciences, Mayo Clinic, Rochester, MN USA; 4 Department of Pediatric and Adolescent Medicine, Mayo Clinic, Rochester, Rochester, MN USA; 5 Division of Epidemiology, Mayo Clinic, Rochester, MN USA

## INTRODUCTION

Recent US data reveal concerning declines in cervical cancer (CC) screening rates and persistent disparities in CC screening and related outcomes by sociodemographic factors.^1,2^ The U.S. Preventive Services Task Force endorsement of HPV testing as a primary approach to CC screening offers an opportunity to explore self-sampling (patient collection of a vaginal swab) as an alternative to clinician sampling.^3^ Self-sampled HPV testing with timely follow-up care has the potential to improve CC screening uptake particularly in low resource settings. With the goal to inform self-sampling interventions to improve HPV testing uptake among underscreened women, this study examines awareness and use of HPV testing and acceptability of self-sampling and differences by sociodemographic factors among CC screening-eligible women living in Minnesota.

## METHODS

### Study Population

Data were from the omnibus 2019 Minnesota State Survey implemented by the Minnesota Center for Survey Research at the University of Minnesota (UMN) through telephone interviews between October 2019 and March 2020, using a simple random sample of Minnesota adult residents with a landline or cell phone (N=612; response rate=10%). Telephone interviewing was abruptly terminated on March 16, 2020, when UMN suspended all on-campus work due to COVID-19. This study was approved by the UMN Institutional Review Board.

### Measures

CC screening-eligible respondents (females ages 21–65 without hysterectomy; N=155) self-reported whether they had heard of HPV testing for CC screening and whether they have had a HPV or Pap test. Respondents were also asked to compare self-sampling for HPV testing to Pap testing done by a clinician in terms of convenience, embarrassment, ease, and pain, and likelihood of following up abnormal results with further testing. Sociodemographic factors including race/ethnicity, education, marital status, household income, housing, and metropolitan area were measured.

### Statistical Analysis

We report descriptive statistics for awareness of HPV testing as a screening option and CC screening history, and acceptance of self-sampling for HPV testing. We examined sociodemographic differences using chi-square test or Fisher’s exact test.

## RESULTS

Table 1 summarizes sample characteristics and HPV testing awareness and self-reported HPV and Pap testing history by sociodemographic factors. Among screening-eligible respondents, 64.5% reported they have heard of HPV testing and 34.7% reported they have had HPV testing, while 89.5% reported they have had Pap testing. Women ages 21–29 (versus 30–65) less frequently heard of HPV testing (*p*=.041) while women ages 21–29 or 40–59 less frequently had HPV testing (*p*=.002). Women without a college degree (versus college graduates) less frequently heard of or had HPV testing (*p*=.014, .015). Additionally, women ages 21–29, racial/ethnic minority (versus non-Hispanic white) women, and women who rent (versus own) their homes less frequently had Pap testing (*p*=.044, .007, .015).

**Table 1:** Awareness of HPV Testing Option and Patient-Reported Cervical Cancer Screening History by Sociodemographic Factors

	Total N (%)	Before today, have you ever heard of using an HPV test for cervical cancer screening a (N=155)		Have you ever had an HPV test to screen for cervical cancer b (N=150) ^c^		Have you ever had a Pap smear or Pap test to screen for cervical cancer (N=153) ^d^	
		N (%) Yes	*p*-value	N (%) Yes	*p-*value	N (%) Yes	*p*-value
Total	155	100 (64.5)		52 (34.7)		137 (89.5)	
Age in years ^e^			.041		.002		.044
21–29	24 (16.8)	9 (37.5)		5 (20.8)		17 (70.8)	
30–39	36 (25.2)	26 (72.2)		18 (51.4)		34 (94.4)	
40–49	35 (24.5)	24 (68.6)		10 (30.3)		32 (91.4)	
50–59	32 (22.4)	18 (56.2)		4 (12.9)		29 (90.6)	
60–65	16 (11.2)	12 (75.0)		9 (56.2)		15 (100)	
Race/ethnicity ^f^			.125		.556		.007
Non-Hispanic white	121 (80.7)	81 (66.9)		42 (35.6)		112 (94.1)	
Other race/ethnicity	29 (19.3)	15 (51.7)		8 (29.6)		22 (75.9)	
Education ^g^			.014		.015		.933
High school or lower	23 (14.9)	9 (39.1)		3 (13.0)		20 (87.0)	
Some college	52 (33.8)	32 (61.5)		14 (28.0)		46 (88.5)	
College graduate	43 (27.9)	31 (72.1)		18 (42.9)		38 (90.5)	
Post graduate	36 (23.4)	28 (77.8)		17 (50.0)		32 (91.4)	
Marital status ^h^			.231		.879		.052
Married	89 (57.8)	62 (69.7)		30 (35.3)		82 (94.3)	
Single	44 (28.6)	26 (59.1)		16 (36.4)		37 (84.1)	
Separated/widowed/other	21 (13.6)	11 (52.4)		6 (30.0)		17 (81.0)	
Household income ^i^			.102		.099		.367
<$30,000	22 (16.9)	8 (36.4)		2 (9.1)		18 (81.8)	
$30,000–$60,000	25 (19.2)	17 (68.0)		10 (41.7)		22 (88.0)	
$60,000–$90,000	31 (23.8)	21 (67.7)		13 (41.9)		26 (86.7)	
$90,000–$120,000	22 (16.9)	14 (63.6)		7 (31.8)		21 (95.5)	
≥$120,000	30 (23.1)	21 (70.0)		10 (35.7)		29 (96.7)	
Housing status ^j^			.257		.990		.015
Own	112 (72.7)	75 (67.0)		37 (34.3)		103 (93.6)	
Rent	42 (27.3)	24 (57.1)		14 (34.1)		33 (78.6)	
Metropolitan area			.068		.389		.284
Greater Minnesota	60 (38.7)	44 (73.3)		18 (30.5)		56 (93.3)	
Twin Cities area	95 (61.3)	56 (58.9)		34 (37.4)		81 (87.1)	

*p*-values obtained from chi-square test or Fisher’s exact test

^a^All participants were given a brief description of the HPV test before answering this question: “The Human Papillomavirus or HPV test is another method used for cervical cancer screening.”

^b^Participants who answered “No” to the question “Before today, have you ever heard of using an HPV test for cervical cancer screening” did not receive this question and were coded as “No” for this question

^c^Missing response=5

^d^Missing response=1

^e^Missing response=12

^f^Missing response=5

^g^Missing response=1

^h^Missing response=1

^i^Missing response=25

^j^Missing response=1

Table 2 summarizes acceptability of HPV testing self-sampling by sociodemographic factors. The majority of respondents reported self-sampling as more convenient (77.8%), less embarrassing (68.6%), easier (74.5%), and less painful (62.7%) compared to Pap testing done by a clinician. Women without a college degree more frequently rated self-sampling as less painful (*p*<.001), while women living in the largely rural greater Minnesota region (versus Twin Cities metropolitan area) more frequently rated self-sampling as more convenient and easier (*p*=.041, .002). Lastly, 94.8% of respondents reported that they would be likely to follow up with further testing upon receiving an abnormal result from the self-sampled HPV test.

**Table 2:** Acceptability of Self-sampling for HPV Testing by Sociodemographic Factors

	Compared to having a Pap test done by your health care provider, collecting your own vaginal sample at home would be… ^a^								How likely would you be to follow up with your health care provider for further testing if you received an abnormal result from your HPV self-sampling ^f^ (N=154) ^g^	
	More convenient (N=153) ^b^		Less embarrassing (N=153) ^c^		Easier (N=153) ^d^		Less painful (N=134) ^e^			
	N (%) Agree	*p*-value	N (%) Agree	*p*-value	N (%) Agree	*p*-value	N (%) Agree	*p*-value	N (%) Likely	*p*-value
Total	119 (77.8)		105 (68.6)		114 (74.5)		84 (62.7)		146 (94.8)	
Age in years ^h^		.423		.791		.675		.614		.309
21–29	21 (91.3)		17 (77.3)		20 (87.0)		15 (68.2)		24 (100)	
30–39	28 (77.8)		23 (63.9)		26 (72.2)		17 (56.7)		32 (88.9)	
40–49	24 (70.6)		26 (74.3)		27 (79.4)		19 (63.3)		34 (97.1)	
50–59	26 (81.2)		24 (75.0)		23 (71.9)		17 (60.7)		31 (96.9)	
60–65	12 (75.0)		11 (68.8)		12 (75.0)		12 (80.0)		16 (100)	
Race/ethnicity ^i^		.271		.166		.692		.318		.621
Non-Hispanic white	97 (80.8)		88 (72.7)		90 (75.0)		63 (61.2)		116 (95.9)	
Other race/ethnicity	20 (71.4)		16 (59.3)		22 (78.6)		20 (71.4)		27 (93.1)	
Education ^j^		.530		.060		.081		<.001		.904
High school or lower	20 (87.0)		19 (82.6)		20 (87.0)		17 (81.0)		21 (95.5)	
Some college	40 (76.9)		39 (76.5)		43 (82.7)		37 (78.7)		49 (94.2)	
College graduate	30 (71.4)		23 (54.8)		27 (64.3)		13 (36.1)		42 (97.7)	
Post graduate	28 (80.0)		24 (66.7)		24 (68.6)		17 (58.6)		34 (94.4)	
Marital status ^k^		.726		.206		.387		.762		.327
Married	67 (76.1)		59 (66.3)		63 (71.6)		49 (62.0)		86 (96.6)	
Single	34 (79.1)		28 (66.7)		33 (76.7)		23 (60.5)		41 (95.3)	
Separated/widowed/other	18 (85.7)		18 (85.7)		18 (85.7)		12 (70.6)		19 (90.5)	
Household Income ^l^		.775		.089		.090		.149		.082
<$30,000	17 (77.3)		15 (68.2)		17 (77.3)		16 (72.7)		19 (86.4)	
$30,000–$60,000	18 (72.0)		18 (75.0)		20 (80.0)		18 (78.3)		25 (100)	
$60,000–$90,000	25 (83.3)		23 (76.7)		22 (75.9)		17 (70.8)		28 (90.3)	
$90,000–$120,000	19 (86.4)		20 (90.9)		21 (95.5)		11 (55.0)		21 (95.5)	
≥$120,000	23 (79.3)		17 (56.7)		19 (63.3)		12 (48.0)		30 (100)	
Housing status ^m^		.170		.516		.752		.773		.685
Own	90 (81.1)		79 (70.5)		84 (75.7)		58 (62.4)		106 (95.5)	
Rent	29 (70.7)		26 (65.0)		30 (73.2)		26 (65.0)		39 (92.9)	
Metropolitan area		.041		172		.002		.201		.263
Greater Minnesota	51 (86.4)		45 (75.0)		52 (88.1)		38 (69.1)		55 (91.7)	
Twin Cities area	68 (72.3)		60 (64.5)		62 (66.0)		46 (58.2)		91 (96.8)	

*p*-values obtained from chi-square test or Fisher’s exact test

^a^Respondents were given a brief description of HPV test self-sampling before answering these questions: “The HPV test to screen for cervical cancer can be done by women in their homes through self-sampling where they are provided a kit with a swab to collect their own vaginal sample.” Questions were measured on a 4-point Likert style scale: strongly agree, somewhat agree, somewhat disagree, strongly disagree. Responses were grouped into two categories: somewhat to strongly agree and somewhat to strongly disagree

^b^Missing response=2

^c^Missing response=2

^d^Missing response=2

^e^ Missing response=21

^f^Question was measured on a 4-point Likert style scale: very likely, somewhat likely, somewhat unlikely, very unlikely. Responses were grouped into two categories: somewhat to very likely and somewhat to very unlikely

^g^Missing response=1

^h^Missing response=12

^i^ Missing response=5

^j^Missing response=1

^k^ Missing response=1

^l^Missing response=25

^m^ Missing response=1

## DISCUSSION

Self-sampling for HPV testing was perceived as more convenient, less embarrassing, easier, and less painful than clinician sampling for Pap testing by most screening-eligible women we surveyed, especially women without a college degree and women living in the greater Minnesota region. Additionally, over 90% of women reported they would seek follow-up testing of an abnormal result from a self-sampled HPV test. These findings suggest that self-sampling has the potential to improve HPV testing uptake for women in medically underserved and rural communities. The disparities by age, education, race/ethnicity, and housing in HPV testing awareness and reported CC screening history confirm previous findings,^4,5^ suggesting interventions promoting HPV testing and self-sampling should focus on underserved populations to reduce existing CC disparities. These data can inform interventions to improve HPV testing uptake among underscreened women through self-sampling.

Limitations of this research include the low response rate, which may introduce bias and limit the generalizability, and the small sample size, which limited statistical power for exploring interaction effects.
